# Quercetin Induces Hepatic Lipid Omega-Oxidation and Lowers Serum Lipid Levels in Mice

**DOI:** 10.1371/journal.pone.0051588

**Published:** 2013-01-24

**Authors:** Elise F. Hoek-van den Hil, Jaap Keijer, Annelies Bunschoten, Jacques J. M. Vervoort, Barbora Stankova, Melissa Bekkenkamp, Laure Herreman, Dini Venema, Peter C. H. Hollman, Eva Tvrzicka, Ivonne M. C. M. Rietjens, Evert M. van Schothorst

**Affiliations:** 1 Human and Animal Physiology, Wageningen University, Wageningen, The Netherlands; 2 Division of Toxicology, Wageningen University, Wageningen, The Netherlands; 3 RIKILT-Institute of Food Safety, Wageningen, The Netherlands; 4 4th Department of Internal Medicine, 1st Faculty of Medicine, Charles University, Prague, Czech Republic; 5 Laboratory of Biochemistry, Wageningen University, Wageningen, The Netherlands; Fundação Oswaldo Cruz, Brazil

## Abstract

Elevated circulating lipid levels are known risk factors for cardiovascular diseases (CVD). In order to examine the effects of quercetin on lipid metabolism, mice received a mild-high-fat diet without (control) or with supplementation of 0.33% (*w/w)* quercetin for 12 weeks. Gas chromatography and ^1^H nuclear magnetic resonance were used to quantitatively measure serum lipid profiles. Whole genome microarray analysis of liver tissue was used to identify possible mechanisms underlying altered circulating lipid levels. Body weight, energy intake and hepatic lipid accumulation did not differ significantly between the quercetin and the control group. In serum of quercetin-fed mice, triglycerides (TG) were decreased with 14% (p<0.001) and total poly unsaturated fatty acids (PUFA) were increased with 13% (p<0.01). Palmitic acid, oleic acid, and linoleic acid were all decreased by 9–15% (p<0.05) in quercetin-fed mice. Both palmitic acid and oleic acid can be oxidized by omega (ω)-oxidation. Gene expression profiling showed that quercetin increased hepatic lipid metabolism, especially ω-oxidation. At the gene level, this was reflected by the up-regulation of cytochrome P450 (Cyp) 4a10, Cyp4a14, Cyp4a31 and Acyl-CoA thioesterase 3 (Acot3). Two relevant regulators, cytochrome P450 oxidoreductase (Por, rate limiting for cytochrome P450s) and the transcription factor constitutive androstane receptor (Car; official symbol Nr1i3) were also up-regulated in the quercetin-fed mice. We conclude that quercetin intake increased hepatic lipid ω-oxidation and lowered corresponding circulating lipid levels, which may contribute to potential beneficial effects on CVD.

## Introduction

Cardiovascular diseases (CVD) are globally the most important cause of mortality. High consumption of fruits and vegetables are thought to be protective against CVD [1]. These protective effects have been suggested to be mediated by the flavonoid content of fruits and vegetables [2]. Various classes of flavonoids are common in plant foods, one being the flavonols. Quercetin is the major dietary flavonol in the Western diet, which is present in, for example, apples, tea, red wine and onions. Epidemiological studies have shown that the intake of this dietary flavonoid is associated with a reduction of CVD risk [3], [4], [5].

Elevated circulating levels of free fatty acids (FFA) and triglycerides (TG) are known risk factors for CVD [6], [7], [8], [9]. In particular, increased levels of FFA and TG are associated with atherosclerosis, ischemic damage, pro arrhythmia, myocardial infarction, and heart failure. Accumulation of toxic lipid intermediates, suppression of glucose usage, or mitochondrial dysfunction potentially play a role in these effects [9]. Several studies showed that supplementation of quercetin to the diet decreased serum FFA and/or TG levels in rodents [10], [11], [12]. The cardio protective properties of quercetin may therefore be explained by the lipid lowering effect of quercetin. However, in these studies the FFA and TG levels were measured with enzyme-based assays. Recently, we have shown that flavonoids interfere with these enzymatic FFA and TG assays, which will lead to incorrect, apparently lower FFA and TG levels [13]. Therefore, it can be questioned whether quercetin has a true biological effect on lipid metabolism. Since various fatty acids are differently associated with CVD risk, it is also important to examine whether quercetin changes specific lipids [14]. Furthermore, based on gene expression analysis some genes have been put forward to explain the effects of quercetin on lipid metabolism [11], [12], but these results are not conclusive. The aim of the present study was to re-examine the effects of quercetin on lipid metabolism, with state-of-the-art analytical techniques, to exclude any interference of quercetin in the measeruments. Gas chromatography (GC) and a novel technique,^ 1^H-nuclear magnetic resonance (^1^H-NMR) lipid profiling of mouse serum (based on [15], [16]), were used to profile and quantify different serum lipids. In addition, whole genome microarray gene expression analysis of liver tissues was applied to unravel the possible underlying mechanisms. For this gene expression analysis the liver was chosen as target organ, since it is one of the major effector organs of lipid metabolism. This principal combination of profiling of serum lipids and gene expression were used to investigate the mechanisms of action of quercetin on lipid metabolism.

**Figure 1:**
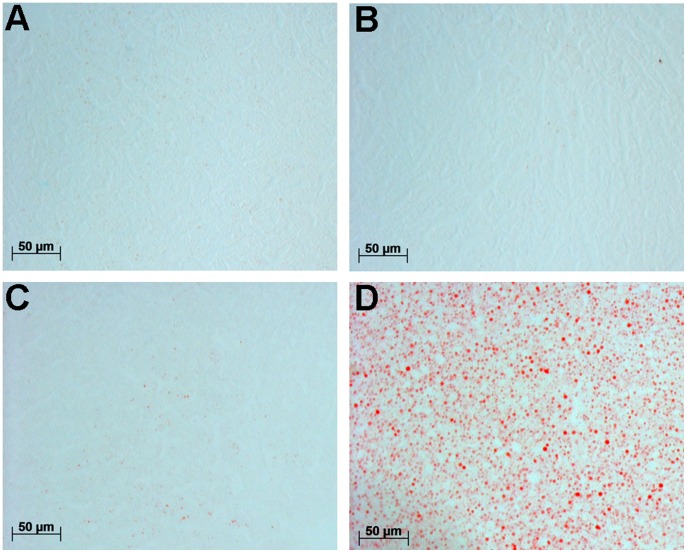
Representative pictures of hepatic lipid staining with Oil red O. There were no significant differences in lipid accumulation between the control (A) and the quercetin (B) group. The lipid levels were comparable to mice fed a normal-fat diet (C) and much lower than the positive control of hepatic lipid accumulation from mice fed a high-fat diet (D).

The results show that a quercetin supplemented mild-high-fat diet in mice increased hepatic lipid metabolism, especially omega (ω)-oxidation and reduced corresponding circulating lipid levels. These results contribute to the understanding of the protective properties of quercetin on CVD.

**Figure 2:**
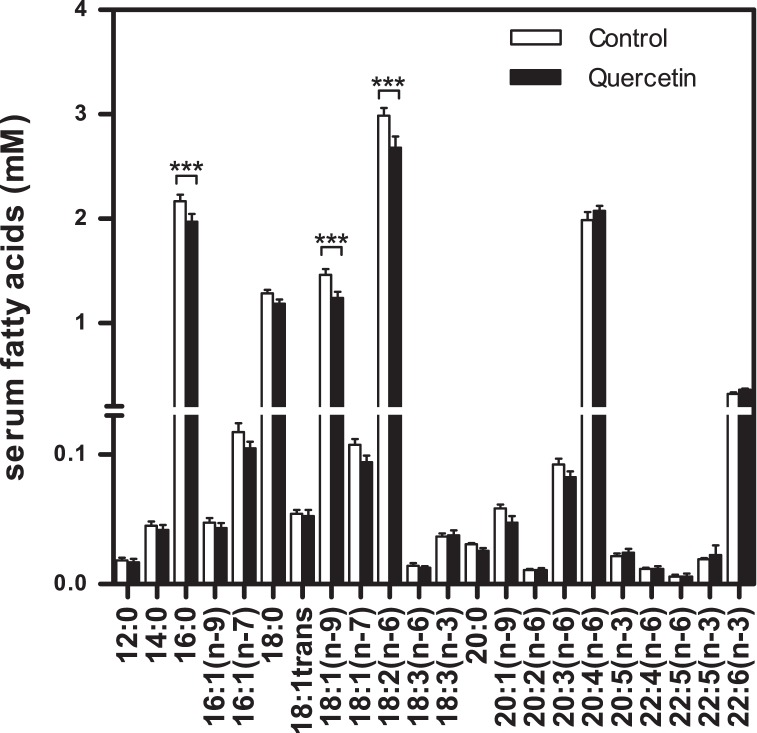
Cumulative serum profile of fatty acids originating from total lipids. Fatty acids were measured with GC. The levels of palmitic acid (16∶0), oleic acid (18∶1(n-9)), and linoleic acid (18∶2(n-6)) were significant lower in the quercetin group. Data is presented as mean ± SEM. Asterisks indicates a significant difference between the control and the quercetin group; *** p<0.001.

**Table 1:** Fatty acid composition of the control and quercetin diet in percentages.

Lipids	Control diet (%)	Quercetin diet (%)
C12∶0	0.09	0.08
C14∶0	1.11	1.09
C14∶1	0.06	0.06
C15∶0	0.08	0.08
C16∶0	22.40	22.26
C16∶1	1.25	1.25
C17∶0	0.30	0.30
C17∶1	0.19	0.15
C18∶0	10.79	10.87
C18∶1 trans	0.46	0.47
C18∶1	33.87	33.94
C18∶1	1.60	1.61
C18∶2	24.23	24.21
C18∶3(n-6)	0.01	0.01
C18∶3	0.71	0.72
C20∶0	0.23	0.22
C20∶1	0.55	0.56
C20∶2	0.29	0.30
C20∶3(n-6)	0.05	0.05
C22∶0	0.06	0.06
C20∶3(n-3)	0.20	0.20
C24∶0	0.06	0.06
C22∶5(n-3)	0.00	0.07
C22∶6(n-3)	0.02	0.03
Saturated FA	35.12	35.02
MUFA	37.98	38.04
PUFA	25.51	25.59

## Materials and Methods

### Animals and Treatments

Twenty-four male C57BL/6JOlaHsd mice (Harlan Laboratories, Horst, The Netherlands) were individually housed and maintained under environmentally controlled conditions (temperature 21°C, 12 h/12 h light-dark cycle, 45% humidity). The mice had *ad-libitum* access to food and water. The food was a pelletized diet (Research Diets Services B.V., Wijk bij Duurstede, the Netherlands) with a mild-high-fat content of 30 energy % (en%). The fat content (en% and fat composition) corresponds to the average human intake in the Netherlands (Dutch Food Consumption Survey, 1998). The mice entered the experiment at 10 weeks of age. After two weeks of adaptation, the quercetin group (n = 12) received the mild-high-fat diet supplemented with 0.33% (w/w) quercetin (Sigma, Zwijndrecht, the Netherlands) for twelve weeks. The control group (n = 12) was given the mild-high-fat diet without quercetin. The body weight and food intake of individual mice were monitored on a weekly basis. After 12 weeks of intervention all mice were fasted for two hours before anesthetisation by inhalation of 5% isoflurane. Blood was sampled via orbital extraction in collect serum tubes (Greiner Bio-one, Longwood, USA), which were centrifuged for 10 min at 3000 g 4°C to obtain serum, which was stored at −80°C. After blood collection, the mice were killed using cervical dislocation and the liver was dissected, weighted and snap frozen in liquid nitrogen and stored at −80°C. The experiment was performed according to the Dutch Animal Experimentation Act (1996) and the experimental protocol was approved by the Animal Welfare Committee of Wageningen University, Wageningen, The Netherlands (DEC 2007080).

**Figure 3:**
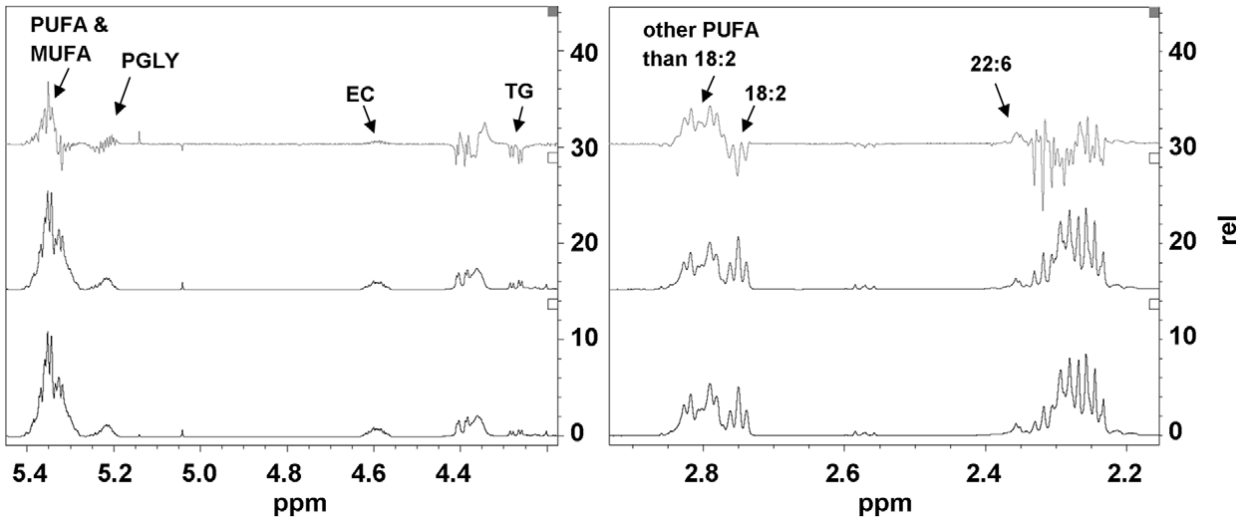
^1^H NMR difference spectrum of the quercetin-fed mice minus the control mice. Serum samples from mice exposed to quercetin minus the ^1^H NMR spectra of the sera from control mice is represented by the top line. The control group is represented by the middle line and the quercetin group is represented by the lowest line. Two representative parts of the spectrum are presented in the figure. PUFA, poly unsaturated fatty acids; MUFA, mono unsaturated fatty acids; FA, fatty acids; TG, triglycerides; PGLY, phosphoglycerides; PC phosphatidylcholine; EC, esterified cholesterol; TC total cholesterol.

### HPLC Analysis of Quercetin Serum Levels

HPLC with a coulometric array detector was used to measure the amount of quercetin in serum. For this 35 µl of serum was hydrolyzed by incubation with 15 µl of 12.5 mg/ml β-glucuronidase/sulfatase in 0.5 M sodium acetate (pH = 5) with 5 g/l ascorbic acid for two hours at 37°C to obtain deconjugated quercetin, isorhamnetin and tamarixetin. Subsequently, all samples were deproteinized by mixing with 100 µL acetonitrile and 50 µL 20% H_3_PO_4_, with 3g/L ascorbic acid and centrifugation for 10 min at 13500 rpm at 5°C. Twenty µl of the supernatant was analyzed on a HPLC system consisting of two pumps (model L-2100; Hitachi, Tokyo, Japan), an autosampler (Model L-2200, Merck Hitachi), a CoulArray Module (Model 5600, ESA, Chelmsford, MA, USA) with electrochemical channels using carbon electrodes arranged in line and set to increasing specified potentials (1 = 20 mV; 2 = 100 mV; 3 = 250 mV; 4 = 500 mV) and a thermostatic column/cell chamber set at 30°C. The chromatography was performed on a Platinum C18 column (EPS; 150×4.6 mm, 3μ, Grace Davison Sciences, Deerfield, IL, USA) equipped with a MPLC Newguard precolumn (Brownlee RP18 7 µm 15×3.2 mm, Perkin Elmer, Shelton, CT, USA), using a gradient elution with two mobile phases. Mobile phase A consisted of 15% acetonitrile in 25 mM H_3_PO_4_ buffer (pH 2.4). Mobile phase B consisted of 50% acetonitrile in 25 mM H_3_PO_4_ buffer (pH 2.4). The gradient, at a flow rate of 1.0 ml/min, was as follows: 0–20 min, linear gradient from 0% to 100% mobile phase B; 20–22 min, isocratic at 100% B; 22–30 min, linear return from 100 to 0% B; the total runtime was 30.0 min. Quercetin, isorhamnetin and tamarixetin were quantified using calibration curves made with commercially available standards.

**Figure 4:**
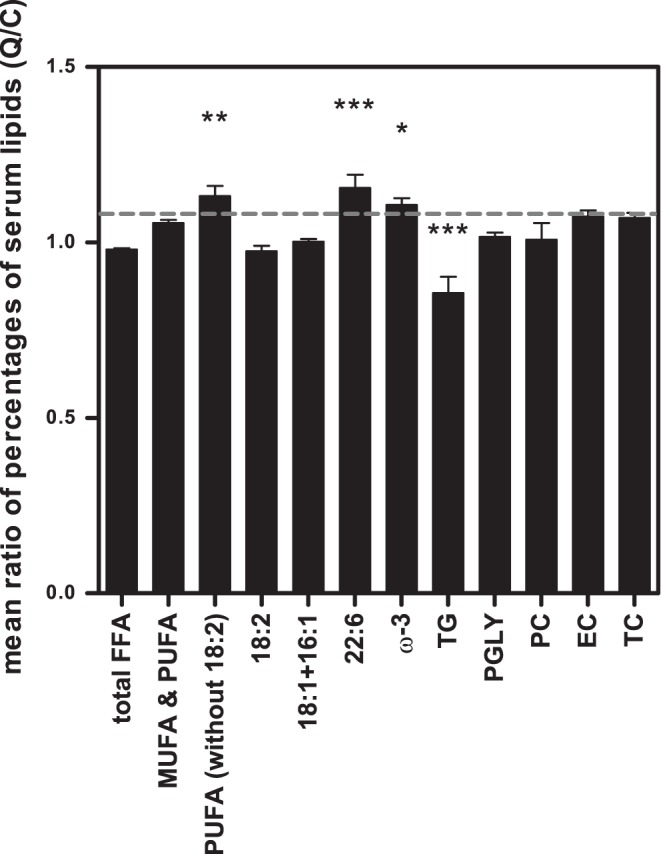
Percentages of lipids present in serum per mouse plotted for quercetin mice to control mice. Lipids were measured with ^1^H NMR. Data is presented as the mean ratio of percentages of lipids present in serum per mouse plotted for quercetin-fed (Q) mice over control (C) mice. Total FFA were not changed, while other PUFA than 18∶2 FA, 22∶6 FA, and, w-3 FA were significantly increased. TG were significantly decreased by the quercetin diet. Data is presented as mean ± SEM. Asterisks indicates a significant difference between the control and the quercetin group; * p<0.05, **p<0.01, *** p<0.001. PUFA, poly unsaturated fatty acids; MUFA, mono unsaturated fatty acids; FA, fatty acids; TG, triglycerides; PGLY, phosphoglycerides; PC phosphatidylcholine; EC, esterified cholesterol; TC total cholesterol.

### Hepatic Lipid Staining with Oil Red O

Frozen liver sections (7 µm) were fixed with 3.7% buffered formalin. Neutral lipids were stained with Oil red O (Sigma). The stained areas were quantified based on a described method [17] using Photoshop software (version 12.0.4, Adobe). Briefly, contrast was enhanced with automatic contrast tool, red pixels were selected with the colour range selecting tool, and total selected area was measured in µm^2^. Ten to 16 pictures per animal were quantified (n = 6–8). Controls of hepatic lipid accumulation were liver of C57BL/6JOlaHsd mice fed a normal-fat diet (10 en% of fat) or a high-fat diet (40 en% of fat) for twelve weeks.

**Figure 5:**
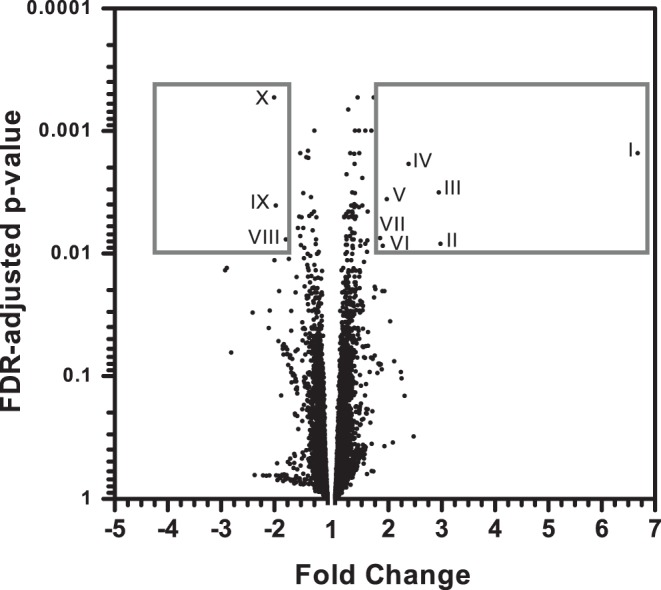
Volcano plot of all expressed probes by global hepatic gene expression analysis. Volcano plot of all probes showing statistics FDR-adjusted p-values plotted against the fold change of each probe (quercetin vs. control). Frames outline genes that are regulated with absolute fold change >1.75 and a FDR-adjusted p-value <0.01; these gene symbols, names and functions are also represented in table 1.

**Table 2:** Regulated hepatic genes with an absolute fold change >1.75 and FDR-adjusted p-value <0.01.

Gene Symbol	Gene Name	Fold Change	FDR adjustedp-value	Function
I. Cyp4a14	Cytochrome P450 4a14	6.68	0.0015	ω-oxidation of medium-chain fatty acids
II. Cyp4a10	Cytochrome P450 4a10	2.98	0.0083	ω-oxidation of medium-chain fatty acids
III. Usp2	Ubiquitin specific peptidase 2	2.95	0.0032	regulation of intracellular protein breakdown, cell cycle regulation and stress response
IV. Acot3	Acyl-CoA thioesterase 3	2.38	0.0018	catalysator of hydrolysis of acyl-CoAs (C12–C16) after ω-oxidation to FFA and coenzyme A
V. Por	P450 (cytochrome) oxidoreductase	1.97	0.0036	electron donor for the microsomal cytochrome P450 mixed-function oxidase system
VI. Cyp4a31	Cytochrome P450 4a31	1.90	0.0086	ω-oxidation of medium-chain fatty acids
VII. Coq10b	Coenzyme Q10 homolog B	1.85	0.0074	an essential electron carrier and proton translocator in the mitochondrial respiratory chain
VIII. Insig2	Insulin-induced gene 2	−1.78	0.0076	lipid and cholesterol metabolic process
IX. Spon2	Spondin 2, extracellular matrix protein	−1.98	0.0040	essential in the initiation of the innate immune response
X. Chka	Choline kinase alpha	−2.00	0.0005	phosphatidylcholine biosynthesis

### Serum Lipid Analysis with Enzymatic Assays

FFA assay (Wako NEFA-HR(2) kit, Sopachem BV, Ochten, The Netherlands) and TG assay (TG liquicolor kit, Human, Wiesbaden, Germany) were performed as described previously [13].

**Figure 6:**
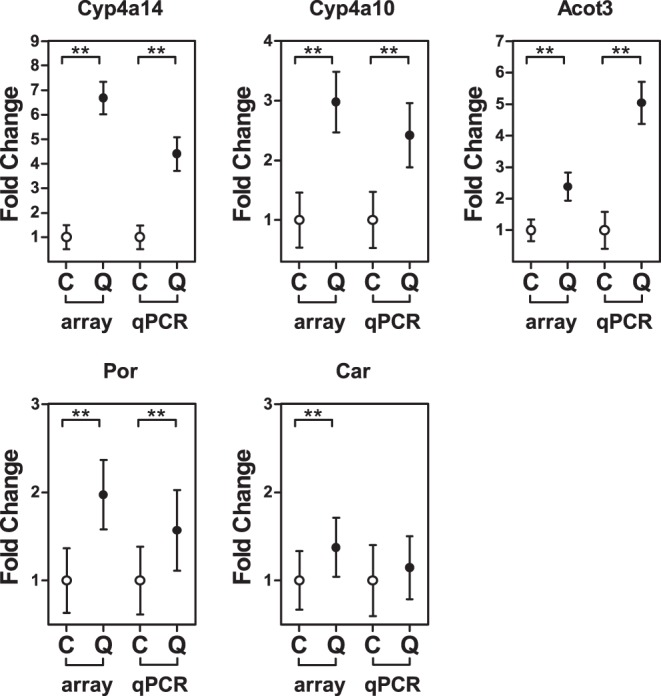
Microarray confirmation by RT-qPCR. The quercetin (Q) regulated genes Cyp4a14, Cyp4a10, Acot3, Car (Nr1i3) and Por, compared to the control (C) found by microarray analysis were confirmed with RT-qPCR. Data is presented as mean ± SEM (n = 12). Asterisks indicates a significant difference between the control and quercetin group; ** p<0.01.

### Serum Fatty Acid Analysis with GC

Total serum fatty acids were extracted from 50 µl serum as described [18], using dichloromethane instead of chloroform [19]. Ten µg of nonadecanoic acid methyl ester (NuCheck Prep, USA) was added to each sample before extraction, as an internal standard. Samples were transmethylated to fatty-esther methyl esthers (FAME) by incubation in 1 M sodium methoxide in dry methanol for 20 min at 80°C. The reaction mixture was then cooled, acidified with 98% sulphuric acid and incubated for 1 hour at room temperature to methylate free acids. Lipid methyl esters were extracted with hexane, and the hexane extracts were subsequently dried under a nitrogen flow. Next, the residue was dissolved in 100 µl of n-heptane and stored at −20°C under nitrogen until analysed. All reactions were performed under nitrogen atmosphere. GC was performed with a Trace-GC gas chromatograph combined with AS 2000 autosampler (ThermoFinnigan, USA), equipped with a capillary split/splitless injector and a flame ionization detector. Analyses of FAME were performed on a fused-silica capillary column coated with chemically bond stationary phase CP-Sil 88 CB (100 m, 0.32 mm I.D.). The oven temperature was programmed as follows: from 80°C to 260°C at 2°C/min, then to 280°C at 10°C/min, where it was maintained for 45 min. The injector and detector temperatures were set at 250°C and 270°C, respectively. Hydrogen carrier gas was maintained at a head pressure of 70 kPa and total flow of 44 ml/min, with a split ratio of 1∶35. Integration software Clarity version 2.4.1.57 (Data Apex Ltd. Prague, Czech Rep.) was used for data acquisition and handling.

**Figure 7:**
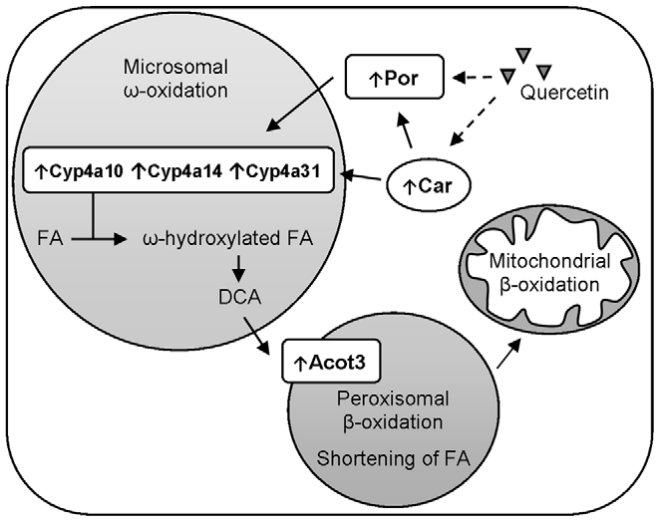
Schematic representation of the quercetin-regulated genes involved in ω-oxidation. Microarray and RT-qPCR analysis showed an up regulation of Cyp4a14, Cyp4a10, Acot3, Por and Car. Quercetin is suggested to activate Car and/or Por (dashed arrow). Activation of the transcription factor CAR can induce the microsomal cytochrome P450 enzymes, CYP4a14, CYP4a10 and CYP4a31, which are important enzymes involved in ω-oxidation. POR is the electron donor for the microsomal cytochrome P450 mixed-function oxidase system. Formed DCA by ω-oxidation are further degraded by peroxisomal β-oxidation to shorter chain fatty acids. ACOT3 is involved in the transport of DCA into the peroxisomes by hydrolysis of long-medium chain fatty acyl-CoA esters to FFA, which can be further transported out of peroxisomes to mitochondria for β-oxidation or excreted in the urine. FA, fatty acids; DCA, dicarboxylic acids.

### Serum Lipid Analysis with ^1^H-NMR

Total serum lipids were extracted from 20 µl of blood serum as described [18] based on [15], [16] with some adaptations to optimise the protocol. Briefly, 20 µl of 150 mM sodium phosphate buffer with 0.04% azide (pH = 3) was added per serum sample. Then 900 µl chloroform:methanol (2∶1) v/v and 900 µl 0.15 M NaCl (pH = 3) were added to the sample. Samples were shaken for 5 minutes on a horizontal shaker and centrifuged for 10 minutes at 4500 g to separate the organic and water phase. The lower organic phase was recovered and the aqueous layer was extracted again with 500 µl chloroform. The collected organic layers were combined and evaporated to dryness using argon. The samples were vacuum and freeze dried and dissolved in chloroform containing 0.03% tetramethylsilane. ^1^H NMR was measured on a Bruker AVANCE spectrometer operating at 600 MHz. For each spectrum 256 (Free induction decays (FID)) transients were collected with a flip angle of 90°, with an acquisition time of 1.82 s, a relaxation delay of 4 s, a spectral width of 30 ppm and a standard noesy 1D pulse sequence, at 25°C, and four dummy scans were used. The FID with 64K data points were once zero-filled and multiplied by an exponential window function with a 0.2 Hz Line-broadening before a subsequent Fourier transformation. To all spectra a baseline correction was applied and the spectra were aligned on the chloroform peak at 7.24 ppm.

The nature of the various resonances was based on the ^1^H NMR spectra as reported [15], [16]. The regions selected to quantify the different lipid fractions were as follows; TG (4.300–4.250 ppm), total FFA (1.380–1.198 ppm), mono unsaturated fatty acids (MUFA) and PUFA (−CH = CH−)(5.41 to 5.27 ppm), other PUFA than 18∶2 FA (2.862–2.768 ppm), 18∶2 FA including linoleic acid (18∶2(n-6)) (2.767–2.721 ppm), 18∶1 and 16∶1 FA including oleic acid (18∶1(n-9)) (2.050–2.011 ppm), 22∶6 FA including docosahexaenoic acid (22∶6(n-3)) (2.379–2.342 ppm), ω-3 FA (0.957–0.947 ppm), phophoglycerides (PGLY) (5.258–5.190 ppm), phosphatidylcholine (PC) (3.787–3.738 ppm), esters of cholesterol (EC) (4.651–4.539 ppm) and total cholesterol (TC) (0.902–0.895 ppm).

### Fatty Acid Composition of Diets

Fatty acids from the diets were extracted with accelerated solvent extraction according to the manufacturer’s protocol (Thermo Scientific) and dissolved in toluene. Subsequently, toluene was evaporated under nitrogen at 40°C and fatty acids were dissolved in iso-octane (5 ml) and 200 µl 2 M KOH in methanol was added and the mixture was shaken for one minute. For neutralization, NaHSO_4_ was added and samples were shaken for 1 minute. The iso-octane fraction (1 µl) was injected in the gas chromatograph equipped with a capillary split injector (split ratio 1∶40) and flame ionization detector. Analyses were performed on a CP select column for FAME (50 m×0.25 mm ID). The oven temperature was programmed from 100°C to 230°C at 6°C/min.

### RNA Isolation

For RNA isolation, liver was homogenized in liquid nitrogen, total RNA was isolated using TRIzol reagent (Invitrogen, Breda, The Netherlands) followed by purification with RNeasy columns (Qiagen, Venlo, The Netherlands). RNA concentration and purity were measured using a Nanodrop spectrophotometer (IsoGen Life Science, Maarsen, The Netherlands); all RNA samples were of high purity. RNA quality was additionally checked on the Experion automated electrophoresis system (Bio-Rad, Veenendaal, The Netherlands).

### Microarray Analysis

For global transcriptome analysis of liver samples, 4×44 K Agilent whole-mouse genome microarrays (G4122F, Agilent Technologies Inc., Santa Clara, CA) were used. Preparation of the samples and the microarray hybridizations were carried out according to the manufacturer’s protocol with a few modifications as described previously [20]. All materials and reagents were from Agilent Technologies, Palo Alto, USA unless stated otherwise. Briefly, cDNA was synthesized for each animal from 1 µg RNA using the Agilent Low-RNA Input Fluorescent Linear Amplification Kit without addition of spikes. Thereafter, samples were split into two equal amounts, to synthesize Cyanine 3-CTP (Cy3) and Cyanine 5-CTP (Cy5) labelled cRNA, using half the amounts per dye as indicated by the manufacturer. Labelled cRNA was purified using RNeasy columns (Qiagen). All samples had a cRNA yield higher than 825 ng and a specific activity of at least 8.0 pmol Cy-dye per µg cRNA. Cy3-labeled cRNA samples were pooled on an equimolar basis and used as a common reference pool. Individual 825-ng Cy5-labeled cRNA and 825-ng pooled Cy3-labeled cRNA were fragmented in 1× fragmentation and 1× blocking agent at 60°C for 30 min and thereafter mixed with GEx hybridization buffer (HI-RPM) and hybridized in a 1∶1 ratio at 65°C for 17 h in the Agilent Microarray Hybridization Chamber rotating at 10 rpm. After hybridization, slides were washed according to the manufacturers’ wash protocol. Arrays were scanned with an Agilent scanner with 10 and 100% laser-power intensities.

### Normalisation and Microarray Data Analysis

Signal intensities for each spot were quantified using Feature Extraction version 9.1 (Agilent Technologies). Median density values and background values of each spot were extracted for both the experimental samples (Cy5) and the reference samples (Cy3). Quality control for every microarray was performed visually by using ‘Quality control graphs’ from Feature extraction and M-A plots and box plots, which were made using limmaGUI in R (Bioconductor, Wettenhal, 2004). Data were imported into GeneMaths XT 2.0 (Applied Maths, Sint-Martens-Latem, Belgium). Spots with an average Cy5 and Cy3 signal twice above background were considered expressed and log transformed. The Cy5 signal was normalized against the Cy3 intensity as described before [21]. Pathway analysis was performed using MetaCore (GeneGo, St. Joseph, Michigan, USA) and Ingenuity Systems (Ingenuity, Redwood City, California, USA). Fold change was expressed as the ratio of the quercetin group versus the control group. Microarray data has been deposited in NCBI Gene Expression Omnibus (GEO) under accession number GSE39140.

### Real Time Quantitative Polymerase Chain Reaction (RT-qPCR)

RT-qPCR was performed using the RNA of the liver samples to validate the microarray data. One microgram of RNA of all individual samples was used for cDNA synthesis using the iScript cDNA synthesis kit (Bio-Rad). RT-qPCR reactions were performed with iQ SYBR Green Supermix (Bio-Rad) using the MyIQ single-colour real-time PCR detection system (Bio-Rad). Individual samples were measured in duplicate. Data were normalized against reference genes beta-2 microglobulin (B2m) and hypoxanthine phophoribosyltransferase 1 (Hprt1) which were chosen based on stable gene expression levels (geNorm, Ghent University Hospital, Ghent, Belgium) and the microarray data. Primers were designed using the NCBI Primer-Blast (NCBI Web site). Sequences of the used primes were as follows: cytochrome P450 4a14 (Cyp4a14); 5′-TTCTTTCGCCTGCGGAATGC-3′ and 5′-CACTCCATCTGTGTGCTCGTGA-3′, cytochrome P450 4a10 (Cyp4a10); 5′-TCTACCCACCTGTCCCAGGC-3′ and 5′-ACACCTCTGGATTTGGCCACA-3′, acyl-CoA thioesterase 3 (Acot3); 5′-GCTGTGACCTACCTGCTCAGTCA-3′ and 5′-ATATAGAGCCATTGATGATGACAGCGG-3′, cytochrome P450 oxidoreductase (Por); 5′-CGAGGGCAAGGAGCTGTACC-3′ and 5′-CACAGGTGGTCGATGGGTGG-3′, constitutive androstane receptor (Car; official gene symbol Nr1i3); 5′-CCGTGTTGCCTCTGCTCACA-3′ and 5′-GGTTAGGGACCGGAAGAGCG -3′, beta-2-microglobulin (B2m); 5′-CCCCACTGAGACTGATACATACGC-3′ and 5′-AGAAACTGGATTTGTAATTAAGCAGGTTC-3′, hypoxanthine-guanine phosphoribosyltransferase (Hprt1); 5′-TGACACTGGTAAAACAATGCAAACTTTG-3′ and 5′-GAGGTCCTTTTCACCAGCAAGCT -3′.

### Statistical Analysis

For microarray analysis, Student’s *t* tests were used with false discovery rate (FDR) adjustment for multiple testing correction according to Benjamini-Hochberg [22]. GraphPad Prism version 5.03 (Graphpad Software, San Diego, USA) was used for other statistical analysis, with Student’s *t* test being used to compare the two groups. Two-way ANOVA was used for analysis of the lipid profiles in serum and diets, followed by a Bonferroni post hoc test. P-values smaller than 0.05 were considered statistically significant.

## Results

### Body Weight, Energy Intake and Quercetin Uptake

Body weight (BW) and energy intake of the adult male mice, which were fed a mild-high-fat diet with or without quercetin supplementation, were not significantly different between the quercetin and the control group during all 12 weeks. Final body weight was 27.9±1.9 and 28.5±1.6 (mean ± SD) gram, and cumulative total energy intake was 654±25 and 661±30 kJ for the quercetin and control group, respectively.

The calculated quercetin intake for the quercetin-fed mice was ∼400 mg/kg BW/day. The sum of quercetin and isorhamnetin after deconjugation in serum was 13.5±3.1 µM expressed as aglycone (quercetin was 6.7±0.9 µM, isorhamnetin was 6.8±2.6 µM, and no tamarixetin was found). No quercetin was found in serum of the control animals.

Relative liver weight was significantly lower in the quercetin-fed mice (3.80% ±0.20; p = 0.007) compared to the control mice (4.08%±0.26), while no significant differences were found for other organs, including white adipose tissue, brown adipose tissue, lung, heart, muscles (data not shown). Hepatic lipid staining showed no significant differences between the quercetin and control group (Figure 1a and b); the Oil red O recorded areas were 480±493 µm^2^ and 321±440 µm^2^, respectively. The hepatic lipid levels were much lower than a positive control of hepatic lipid accumulation (13,151±4,410 µm^2^) and in the same range of hepatic lipid levels found in liver of mice fed a normal-fat diet (516±271 µm^2^) (Figure 1c and d).

### Serum Lipids as Determined by Enzymatic Assays

Quantification of serum FFA and TG levels was performed using the enzymatic FFA and TG assays, which showed a significant decrease of 13% FFA (p<0.05) and 27% TG (p<0.05) due to the quercetin diet. However, since quercetin has been shown to interfere with these enzymatic assays resulting in incorrect, apparently lower FFA and TG levels [13], two additional analytical techniques were applied to assess serum lipid profiles, and to check if the decreased FFA and TG levels detected by the enzymatic assays represent real biological effects.

### Serum Fatty Acid Profile as Determined by GC

GC fatty acid profiles reveales fatty acids originating from TG, FFA, cholesteryl esters and phospholipids. The serum fatty acid profile showed a total decrease of 7% (p<0.001) in the quercetin-fed mice. The levels of palmitic acid (16∶0), oleic acid (18∶1(n-9)) and linoleic acid (18∶2(n-6)) were 9–15% lower (p<0.001) in the quercetin group (Figure 2). These are the main fatty acids in the quercetin diet and the control diet, which were similar in terms of fatty acid composition (Table 1). All other fatty acids that were present in the serum showed a tendency of decreased levels due to the quercetin treatment, except for some poly unsaturated fatty acids (PUFA), such as arachidonic acid (20∶4(n-6)) and docosahexaenoic acid (22∶6(n-3)) which were slightly, but non significantly, increased in the serum of quercetin-supplemented mice.

### Serum Lipid Profile as Determined by ^1^H NMR

^1^H NMR measurement reveals total TG, FFA, cholesterol and phospholipids that are present in serum, separately. Figure 3 presents the ^1^H NMR difference spectrum, that is composed of the ^1^H NMR spectra of serum samples from mice exposed to quercetin (n = 12) minus the ^1^H NMR spectra of the sera from control mice (n = 12). The different regions (based on [15], [16]) were selected to obtain information on several subsets of FFA and/or TG, as shown in Figure 3. Integration of the respective peak areas in the ^1^H NMR spectra of the individual serum samples resulted in the amounts of the various lipids. The data are presented in figure 4 as the mean ratio of percentages of lipids present in serum of quercetin-fed mice as compared to control mice. From these data it follows that upon quercetin exposure the levels of TG are significantly decreased with 14% (p<0.001), while some specific poly unsaturated FFA levels were increased with 11–16%; these were PUFA other than 18∶2 FA (p<0.01), 22∶6 FA (p<0.001), and ω-3 FA (p<0.05). The total amount of FFA was the same in both groups, the levels of PGLY and PC showed no change and the EC and TC showed a slight increase, although not significant. This implies that the overall decrease in lipid levels that are observed in the GC analysis are due to a decrease in TG.

### Quercetin Altered the Expression of Genes Involved in Lipid Metabolism

Gene expression was analysed using whole genome gene expression microarrays. Of the 23,256 probes being expressed, 415 probes were significantly differently expressed by quercetin treatment (p<0.05, FDR-adjusted). Regulation of lipid metabolism by quercetin was found by pathway analysis of the differently expressed genes using the two major analysis programs, Metacore and Ingenuity. ‘Phospholipid metabolism’ was the most significantly regulated pathway in Metacore with a p-value of 4.8E-05 (with 3 down-regulated and 1 up-regulated gene out of 33 genes). Ingenuity pathway analyses identified: ‘LPS/IL-1 Mediated Inhibition of RXR Function’ as the most regulated pathway (p-value of 5.46 E–05, with 13 up-regulated genes out of 187). In this pathway Car is the central transcription factor and the genes, in particular cytochromes P450, overlap partly with the ‘linoleic acid pathway’, which is the number 3 pathway (p-value of 1.7E–03 with 4 up-regulated genes out of 83 genes) in Metacore. Although, in each of these pathways a relative small number of genes were regulated out of the total number of genes present, it was clear that these regulated genes corresponded with the top significantly regulated genes. The ten most regulated genes (absolute fold change >1.75 and a FDR adjusted p-value <0.01), where almost all involved in lipid metabolism, particularly in ω-oxidation of fatty acids (Figure 5, table 2). These genes involved in ω-oxidation included Cyp4a14, Cyp4a10, Cyp4a31, Acot3 and Por. Altogether, lipid metabolism, and in particular ω-oxidation, were identified as being regulated by quercetin in the liver.

### Confirmation with RT-qPCR

The quercetin induced changes in expression of Cyp4a14, Cyp4a10, Acot3, Car, and Por that were identified by microarray analysis, were confirmed with RT-qPCR (Figure 6). Cyp4a14, Cyp4a10, Acot3 and Por were significantly up-regulated in the quercetin group, while the up regulation of Car followed the same trend, but did not reach significance.

## Discussion

This study showed that chronic intake of quercetin in mice lowered serum lipid levels which are risk factors for CVD. Microarray analysis indicated that hepatic genes involved in lipid metabolism, in particular in ω-oxidation of fatty acids, could be responsible for these quercetin-induced effects.

Other studies have also observed that supplementation of quercetin to a high-fat diet decreases serum FFA and/or TG levels in mice [10], [11], [12]. However, these circulating FFA and TG levels were measured with commercial enzymatic assays, which have recently been found to be sensitive to interference of quercetin and its major metabolite quercetin-3-O-glucuronide, resulting in apparently incorrect lower detected levels [13]. Here, besides these enzymatic assays, we also used two independent analytical methods for quantification of serum lipid profiles; GC and ^1^H NMR techniques. The observed effect of quercetin on lipid levels measured with the enzymatic FFA and TG assays (FFA -13% and TG -27%) was higher than measured with the two analytical techniques (GC: total fatty acids -7% and ^1^H NMR: FFA -2%, TG -14%). This confirms interference of quercetin in the enzyme based assays [13] in the physiological range of quercetin exposure and as a consequence overestimate the lipid lowering effect of quercetin. Nevertheless, with GC and ^1^H NMR a significant reduction in serum lipid levels was found, proving that lipid lowering is a real biological effect of quercetin. The GC data revealed that the specific serum fatty acids palmitic acid (16∶0), oleic acid (18∶1(n-9)) and linoleic acid (18∶2(n-6)), originating from total lipids, were all significantly decreased in the quercetin-fed mice. Moreover, with ^1^H NMR, serum lipids were measured separately, which revealed that serum TG levels of the quercetin group were significantly decreased, while total FFA, cholesterol and phospholipid levels remained unchanged. This indicates that the decreased levels of palmitic acid (16∶0), oleic acid (18∶1(n-9)) and linoleic acid (18∶2(n-6)) found by GC originated from TG. Moreover, the ^1^H NMR data showed unchanged levels of total FFA and increased levels of PUFA in the serum of the mice on the quercetin diet, which indicate a shift from saturated fatty acids to PUFA, which are known as the more healthy fatty acids. Together, these data proved that quercetin significantly reduced serum lipid levels and resulted in a more beneficial lipid profile.

The increased levels of PUFA and the decreased levels of saturated fatty acids cannot be fully explained by the microarray data. Genes involved in beta oxidation or specific desaturases were not differentially regulated by the quercetin diet.

There were no significant differences found in the serum phospholipid levels, while pathway analysis revealed phospholipid metabolism as a regulated pathway. However, based on gene expression it was not clear how phospholipid metabolism would be affected, since up as well down regulated genes were observed in different parts of this pathway, and a relative small number of genes of the total pathway was regulated. Therefore, it was concluded that this was not a crucial pathway in this study. Quercetin induced a decrease in relative liver weight in our study. This decrease cannot be explained by a decrease in hepatic lipid accumulation, because hepatic lipid levels were not affected by quercetin. Other studies [11], [12] have shown a decrease in lipid accumulation in liver upon dietary administration of quercetin and thus seem to be in contrast with our study. While a study with mulberry leaves, high in quercetin, did report unmodified lipid accumulation in the liver [23] and is thus in line with our data. The differences may be explained by the diets used in the different studies. We have used a mild-high-fat diet rich in unsaturated fatty acids, which did not result in extensive lipid accumulation in the liver, since the found hepatic lipid levels were in the same range as found for mice fed a normal-fat diet. The other studies that show a quercetin induced decrease in lipid accumulation used a high saturated fatty acid rich diet which induced lipid accumulation in the liver [11], [12]. This suggests that quercetin may prevent lipid accumulation in the liver under adverse dietary conditions, but not with relatively healthy diets. In general, quercetin induced altered lipid metabolism on a mild-high-fat diet (our study), a normal-fat diet [10], and different high-fat diets [11], [12]. Suggesting, that quercetin can affect lipid metabolism independent of the diet, although the impact of this effect can be different.

Using whole genome microarrays and confirmation by RT-qPCR, we showed that quercetin up-regulates Cyp4a10, Cyp4a14, Cyp4a31, Acot3, Por, and, possibly Car. An integration of these genes into a single ‘hepatic pathway’ differentially expressed by quercetin treatment is proposed in Figure 7. Normally, fatty acids are mainly metabolized by β-oxidation first in peroxisomes (very long chain FFA) and subsequently in mitochondria (long, medium, and short chain FFA). Another type of fatty acid oxidation is ω-oxidation, which occurs in the endoplasmatic reticulum by members of the cytochrome P450 4A family [24]. Omega-oxidation becomes more important during periods of increased influx of fatty acids into the liver, for example in our high-fat diet mice study, in obesity, and when the mitochondrial oxidation system is insufficient to metabolize fatty acids [25], [26]. In these situations ω-oxidation can prevent lipid toxicity [27]. Fatty acids oxidized by ω-oxidation result in ω-hydroxy fatty acids which are then dehydrogenated to a dicarboxylic acid in the cytosol. These dicarboxylic acids are further degraded by peroxisomal β-oxidation to shorter chain dicarboxylic fatty acids, which can be excreted in the urine, metabolized by the peroxisomal oxidation system to succinate and acetyl CoA, or completely oxidized after transport into the mitochondrial β-oxidation system [28]. A small increase of ketone bodies was found in the quercetin-fed mice suggesting an increase of β-oxidation (292.5±199.2 versus 185.6±118.1 µM, p = 0.12).

Acot3 was also up-regulated in our study, and the enzyme ACOT3 hydrolyses long-medium chain fatty acyl-CoA esters to FFA, and thus facilitate transport into peroxisomes. The FFA can subsequently be transported out of peroxisomes to mitochondria for further β-oxidation [29], [30].

It has been described that, among others, palmitic acid (16∶0) and oleic acid (18∶1(n-9)) can be hydroxylated by CYP4A11, the human variant of murine Cyp4a10 [31]. This is especially consistent with the serum fatty acid profile obtained in the present study (Figure 2), where levels of palmitic acid (16∶0) and oleic acid (18∶1(n-9)) were significantly lower in the quercetin-fed mice. The significant up regulation of Cyp4a10, Cyp4a14, Cyp4a31 and Acot3 therefore explains the observed reduced serum levels for these specific fatty acids.

In humans, various polymorphisms are described in the genes of cytochromes P450s and they can be considered as one of the major determinants of individual susceptibility to CVDs [32]. Allelic variations in CYP4A11 are suggested to result in an increased risk for hypertension [25], [32]. Hypertension can be caused by increased serum lipid levels [6], which were decreased by quercetin in our study with concomitant up regulation of Cyp4a genes.

The up regulation of the Cyp4a genes is consistent with the significant, 1.97 fold up regulation of Por by quercetin. POR is an enzyme that is required for electron transfer to cytochrome P450 enzymes and is therefore rate limiting for P450 enzymes. Deletion of the Por gene in a mouse model reduced hepatic P450 activity by more than 95%. Moreover, hepatic Por knockout (Por-KO) mice showed decreased CYP4A protein levels, and an enlarged and fatty liver. Based on these observations, it was concluded that the P450 system plays a major role in regulating lipid homeostasis and hepatic lipid levels [33], [34]. Two to three-fold more genes were significantly regulated when WT mice were exposed to quercetin compared to Por-KO mice. These genes were, among others, involved in fatty acid metabolism pathways. This suggests that hepatic POR mediates many of the biological effects of quercetin, including fatty acid metabolism [35]. These results underscores our data, which showed an up regulation of Por.

It is also suggested that P450 expression can be mediated via a CAR-dependent signaling pathway [36]. CAR is a transcription factor that is highly expressed in the liver. It is shown that ligand dependent activation of CAR increased lipid metabolism in rodents [37], [38] and it is also shown that this can lead to specifically increased expression of genes involved in ω-oxidation [39]. Furthermore, exposure of quercetin to HepG2 cells transfected with CAR showed that CAR can be activated by quercetin [40], [41]. Our data showed significant up regulation of Car (FC = 1.37, FDR adjusted p-value = 0.005), which suggests that Car has an important role in quercetin mediated regulation of lipid metabolism.

This study used male mice, therefore caution is needed in translating these data to female mice. It is known that there are sex differences in the sensitivity to CAR activators and also Cyp4a genes can be under sex-dependent control [42], [43]. In conclusion, quercetin can affect hepatic lipid metabolism, especially ω-oxidation. This is shown by the up regulation of Cyp4a10, Cyp4a14, Cyp4a31, Acot3, Por and the transcription factor Car. These effects are associated with decreased corresponding circulating lipid levels, which may contribute to potential beneficial effects on CVD.
