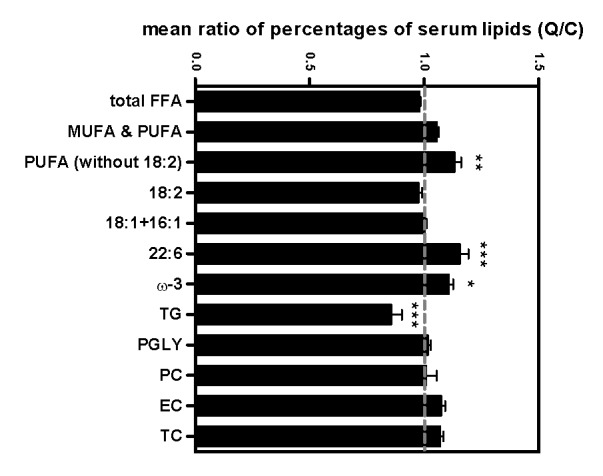# Correction: Quercetin Induces Hepatic Lipid Omega-Oxidation and Lowers Serum Lipid Levels in Mice

**DOI:** 10.1371/annotation/53e96376-38fe-40e7-b73a-60e7232cef0e

**Published:** 2013-09-19

**Authors:** Elise F. Hoek-van den Hil, Jaap Keijer, Annelies Bunschoten, Jacques J. M. Vervoort, Barbora Stankova, Melissa Bekkenkamp, Laure Herreman, Dini Venema, Peter C. H. Hollman, Eva Tvrzicka, Ivonne M. C. M. Rietjens, Evert M. van Schothorst

The dashed guideline in Figure 4 was printed out of place. You can find the corrected Figure 4 here: 

**Figure:**